# Activated Porous Carbon Fiber: New Adsorbent for Sampling and Analysis by Thermal Desorption of Siloxanes in Biogas and Biomethane

**DOI:** 10.3390/ijerph191710890

**Published:** 2022-09-01

**Authors:** Enrico Paris, Pasquale Avino, Ettore Guerriero, Beatrice Vincenti, Adriano Palma, Monica Carnevale, Paolo Benedetti, Marco Torre, Francesco Gallucci

**Affiliations:** 1 Council for Agricultural Research and Economics (CREA), Center of Engineering and Agro-Food Processing, via della Pascolare 16, Monterotondo, 00015 Rome, Italy; 2 Department of Agricultural, Environmental and Food Sciences (DiAAA), University of Molise, via de Sanctis, 86100 Campobasso, Italy; 3 Institute of Atmospheric Pollution Research, National Research Council, Rome Research Area-Montelibretti, Monterotondo, 00015 Scalo, Italy

**Keywords:** pollutant monitoring, activated porous carbon fiber, biomethane quality

## Abstract

The growing global energy demand requires the continuous development and optimization of the production of alternative energy sources. According to the circular economy approach, waste conversion into biogas and biomethane represent an interesting energy source. The input into the distribution network and energy conversion systems of biomethane requires quality monitoring and the use of cleaning up systems. Therefore, there is a need to constantly invest in the development of sampling and analysis systems that save time, costs, and materials. The purpose of this study was to use activated porous carbon fiber (APCF), an extremely versatile material for sampling and analysis by thermal desorption, to show the advantages it has over the adsorbents traditionally used for siloxane monitoring. Siloxanes are among the contaminating compounds that are mainly present in biogas and biomethane, and if not removed sufficiently, they endanger the quality and use of the gas. These are highly harmful compounds since during combustion, they produce quartz particles that are abrasive to the surfaces of the materials involved in the energy production process. In addition, siloxanes directly hinder the energy properties of biomethane during combustion, due to their radical scavenger properties. In this work, the efficiency of APCF tube was evaluated by comparing it with common multilayer tube thought sampling and analyzing siloxanes in lab scale and in real scale (biogas plant). Thermal desorption analysis coupled with GC-MS for the determination of siloxanes showed that the use of APCF allows to obtain better performance. This allows to deduce that APCF is an innovative material for the establishment of a better sampling and analysis method than the current ones, enabling better results to be achieved in the process of monitoring fuel quality in biomethane production and storage facilities.

## 1. Introduction

Siloxanes are a class of organometallic compounds characterized by Si-O-Si structure with organic radicals bound to Si including methyl, ethyl, and other functional organic groups. To date, their annual production worldwide is estimated at over two million tons [1]. Such high demand on the market is due to some of their peculiar characteristics such as solubility in water and in many organic solvents, high thermal stability, and low viscosity and flammability [2]. Siloxanes usually present in biogas are man-made compounds containing silicon and oxygen with organic side groups (methyl groups), called methylsiloxanes [3].

Biodegradable and relatively non-toxic compounds, they are used in various industrial processes and are generally added to consumer products, such as detergents, shampoos, cosmetics, care products (i.e., creams, lipsticks, and deodorants), sealants, paper coatings, and textiles, and are often also used as food additives [4]. This means that siloxanes are abundant in the sewage sludge and consequently lead to the contamination of the biogas and biomethane produced. The level of siloxane is worthy of technical concerns for subsequent damage to combustion engines [5]. The siloxanes in the gas are not reactive or corrosive as such, but they are transformed into hard microcrystalline silica, which has properties like those of glass and leads to abrasive phenomena in the combustion chamber of the engine. They also form a paint coating on all engine surfaces in contact with the oil and, therefore, can alter the finish of the oil retention surface of the cylinder coatings. Such a problem is clear in biogas-powered spark-ignition engines, whose high speeds and working temperatures accentuate these issues [6,7,8]. Biomethane can also be used in fuel cells and again, silicon compounds also have a poisoning effect on the anodic side of the solid oxide fuel cell and can form a scale on the surface of devices such as turbines, thereby reducing their working efficiency [6,8].

Cyclic siloxanes are designated with the letter D, whereas the linear compounds are designated with the letter L or with the M-nomenclature and are named according to the number of Si atoms present in their molecular structure [6].

The siloxanes in biogas are mainly represented by volatile methylsiloxanes (VMS), such as hexamethylcyclotrisiloxane (D3), octamethylciclotetrasiloxane (D4), decamethylcyclopentasiloxane (D5), dodecamethylcyclohexasiloxane (D6), hexamethyldisiloxane (L2), and octamethyltrisiloxane (L3) [9,10,11,12,13,14]. They have relatively low molecular weights, about 450, and are oily colorless liquids at ambient temperature, except for D3, which is a solid. Generally, their higher vapor pressure values suggest that they volatilize earlier, in contrast with the lower vapor pressure of D6 that remain in sludge [15,16]. These observations could explain the lower content of L2, L3, D3, and D6 in biogas, respective to D4 and D5 [11], that are most dominant in biogas (the main chemical-physical characteristics of the siloxanes tested in this paper are summarized in Table 1). Their impact on environment organisms’ health is related to growth of pollution because of their physical properties [17,18,19,20]. Indeed, for example, during biogas combustion, the presence of siloxanes produces silicon dioxide particles causing abrasion and erosion of equipment reducing the process efficiency [21,22]. Their presence can cause corrosion that leads to the wearing out of metallic parts of appliances and engines and a slight deposition in engines cause scaling and damage to the application equipment [23]. Their trace concentrations have been demonstrated to damage gas processing plant because of their oxidation to fine and silica particles during combustion [24]. To ensure tolerance for the engine, including the use of highly corrosion-resistant materials may be an expedient, but the high cost of such materials limits their use [23].Therefore, it is prudent to eliminate contaminants before it is used in engines. The chemical reactions of VMSs oxidation, where the final product is silicon oxide (IV), are [3]:

1. C_8_Si_3_O_2_H_24_ + 16 O_2_ → 3 SiO_2_ + 8 CO_2_ + 12 H_2_O (linear VMS—L3)
2. C_10_Si_5_O_5_H_30_ + 20 O_2_ → 5 SiO_2_ + 10 CO_2_ + 15 H_2_O (cyclic VMS—D5)

The importance of monitoring siloxanes has increased contemporary to the growth of the biogas-to-energy purposes, and its analysis is necessary for the cleaning process before the combustion of biogas [25], even if they are among the most difficult trace compounds to identify and determine. According to Khan M.U. et al., in biogas, the siloxanes concentration is generally between 0 and 40 mg/m^3^ [26] even if in other studies, their concentrations are estimated to beabout 60 mg/m^3^ [11,27]. However, in accordance with Piechota [28], the limit of silicon content (Si) for biogas used in the CHP units is <2 mg/m^3^ and for VMS in biomethane injected to gas grids is 0.05 ± 0.02 mg/m^3^. At present, there is no standard method for the analysis of volatile siloxanes in a gaseous matrix [18]. Several authors have studied this regulatory condition, having no relevant response. For siloxane analysis in biogas, Hagmann et al. [21] used the method with GC–MS, gas chromatography coupled with mass spectrometry, and Huppmann et al. [12] used gas chromatography coupled with flame ionization detection (GC-FID). Most commercially available removal methods are based on adsorption aiming at removing corrosive compounds. To extract siloxanes from biogas, the same materials as XAD resins, activated carbon, polyurethane foam, and tetradecane were tested and for siloxanes sampling, Schweigkofler and Niessner used vacuumized stainless steel canisters [15]. The most common and efficient sampling and analysis techniques include sampling canisters, Tedlar^®^ bags, sorption tubes, and impingers [29]. Several approaches in the literature have involved the use of solid sorbents designed as a siloxane trap [30] subsequently shipped for extraction and analyzed by gas chromatography (GC) coupled with flame ionization detector (FID), atomic emission detector (GC-AED), or mass spectrometer (GC-MS) [10,21,22,30,31]. There is a fair number of studies on the feasibility of different filter and adsorption materials, such as activated carbon, aluminum oxide, ion exchange resins, natural clay minerals, silica gel, molecule sieves, and zeolites [6,16,32,33]. A field-proven technology exists for activated carbon and silica gel which has good affinity to siloxane, but the drawback for purification has low affinity to sulphureous compounds [32]. Some studies include the use of membranes, scrubbing with organic solvents, chelate and polymers solutions, and cryogenic processes. The risk for contaminant breakthrough with activated carbon filtration [4] has enhanced the development of siloxane cleaner processes [34]. Biogas purification has a relevant impact on the economic and energy efficiency of a biogas production facility. Linked to such reasons, the aim of this work is the monitoring and analysis of siloxanes in biogas and biomethane by experimenting, as a sampling and analysis technique, the use of an innovative material: Activated Porous Carbon Fiber (APCF). To the best of the authors’ knowledge, there are no literature studies on the use of activated porous carbon fibers for siloxanes monitoring in biogas and biomethane. An interesting study curried out by Piechota [3] compared four different techniques for sampling siloxanes and subsequently determined in GC/MS: impinger, micro-impinger, TedlarBag, and active-carbon (AC) tube were used. In the cited paper, impinger and micro impinger yielded better results than using activated carbon tubes. Although the use of AC tubes may seem like a similar process to the one tested in this paper, there are substantial differences between AC and APCF. The first is granular and has a surface area usually of about 1000 m^2^/g while the second is fibrous and has a surface area of 2000 m^2^/g. In addition, solvents were used in order to chemically desorb tubes, while in this study thermal desorption was applied. This has resulted in costs and materials savings and in less sample manipulation. This paper shows how the use of APCF with thermal desorption technique coupled to GC-MS represents a step forward compared to the adsorbents currently on the market in terms of precision and accuracy of analysis. This study represents an innovative approach and lays the foundation for future research in this area, comparing this technique and its several advantages with other techniques tested in the literature.

## 2. Materials and Methods

### 2.1. Instrumental Apparatus

The tests were conducted in the LASER-B laboratory of CREA-IT (Monterotondo, Rome). A thermal desorber TD-100xr (Markes) coupled to a GC-MS system (Agilent GC 7890A and MS/MS 7000) was used for the analysis. These parameters were optimized for analytes studied starting from those used in previous work for the analysis of volatile compounds in TD-GC/MS [35]. The optimized parameters used are summarized in Table 2.

### 2.2. Sampling Tubes and Focusing Trap: Assembling and Test

The tubes were assembled using standard empty stainless-steel tubes (Labtech s.r.l. (Rome, Italy) packed with APCF (Labtech s.r.l., characteristics are shown in Table 3). The APCF used had a surface development of 2000 m^2^ g^–1^ and a quantity of 0.180 ± 0.007 g was used for each tube.

After the tubes packing phase with APCF, a conditioning phase is needed. Such a phase has the aim of eliminating extraneous compounds adsorbed on APCF during tubes production, storage, and packing. Conditioning was carried out via a helium flow at 100 mL min^–1^ per 3 h at 400 °C. Subsequently, tubes were sealed with the special caps in brass and nut in Teflon (Swagelok Company, Solon, Oh, USA) and stored inside hermetic containers also containing a vial of adsorbent material to protect them from contaminants during storage.

To produce the focusing trap, an empty trap (cold trap “empty”, Markes) was used. Manufacturers recommend, during packing, using a 0.5 cm section of quartz wool on top of the cold trap (to prevent the adsorbent material from penetrating inside the heated valve) and the adsorbent bed does not exceed 6 cm. With these parameters, the focusing trap was packed with 0.048 ± 0.001 g of APCF (Labtech). Once conditioned, it was introduced inside the thermal desorber, sealing tests were conducted and passed.

To assess tube and focusing trap efficiency both in the adsorption and desorption phases, a double type of test was performed.

Scanning electron microscopy was performed by Supra 25 (Zeiss, Oberkochen, Germany) to determine samples morphology. The samples were coated with 10 nm of conductive material (gold) to prevent charge buildup on specimen surface. The metal was applied in a controlled manner by a sputter coater (Automatic Sputter Coater, Agar Scientific, Stansted, UK).

#### 2.2.1. Adsorption Phase

**Figure 1:**
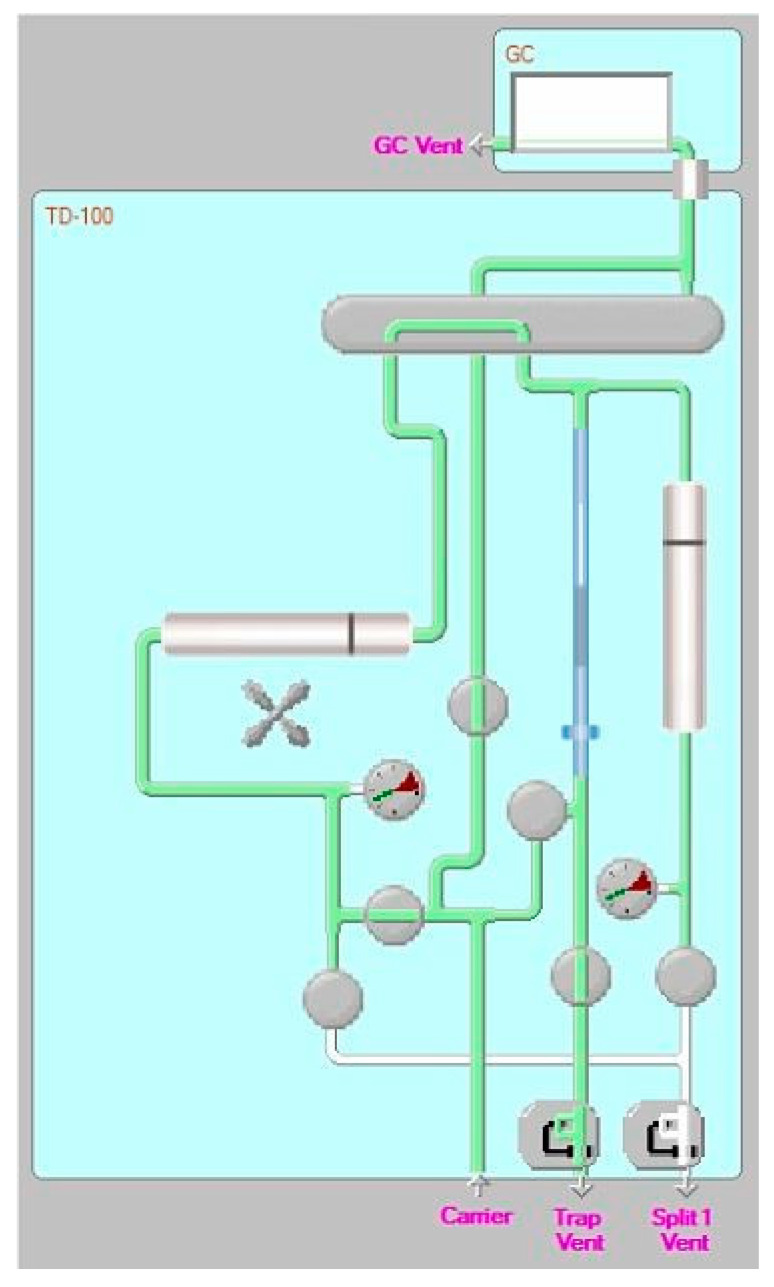
Operating diagram of the thermodesorber. The gas carrier flows to desorb the analytes from the tube (on the left) and focus them on the focusing trap (in light blue). The carrier is then ejected from the trap vent output.

APCF tubes were sampled with different concentrations of siloxanes standard solution (Ultra Scientific Italia) composed by octamethylcyclotetrasiloxane (D4), decamethylcyclotetrasiloxane (D5), and dodecamethylcyclotetrasiloxane (D6) (as already mentioned, three of the most common contaminants in biogas and biomethane) and subsequently desorbed. In this phase, a rather wide and high concentration range of standard solution was adsorbed on APCF tubes (up to 50 ng). Even if it is an excessive concentration for the usually sampled quantities on real plant (between 50 mL and 1000 mL), such concentrations allow to evaluate the solidity of the proposed method even under extreme conditions. During the tube desorption phase (when the analytes are eluted from the sampling tube and focused on the focusing trap), a “backup tube” was placed downstream of the system on the trap vent output. This sampling aimed to evaluate the efficiency of “entrapment” of the focusing trap. In fact, if the analytes taken from the tube could not be collected quantitatively on the focusing trap or had to be transported away for breakthrough effect, they would be expelled from the trap vent output and then sampled from the backup tube (Figure 1).

#### 2.2.2. Desorption Phase

After the tube desorb phase, the cold trap was desorbed three consecutive times. All the sampled analytes were detected only in the first race, while the subsequent desorbing showed no cold trap “memory effect” (the phenomenon that if the focusing trap does not completely desorb, it continues to show previously adsorbed analytes). APCF tubes were also desorbed three times to evaluate the memory effect.

The calibration lines for the D4, D5, and D6 were subsequently produced with a lower concentration range and more coherence with those usually found in biogas and biomethane for the sampled volumes. Five calibration points were interpolated with quantities of 1, 2, 3, 5, and 6 ng on each APCF tube.

### 2.3. Biogas Sampling

In order to assess the APCF packed tubes behavior, such devices were compared with commercial multi-layer tubes packed with a combination of graphite granular carbons (Carbograph 1TD and Carboxen 1003) during a sampling campaign on a biogas production plant. Sampling was carried out through appropriate sampling points (biogas conduit derivations with small quarter-inch flanges) interfaced with adsorbent sampling tubes. Active samples were made, that is using a suction pump and a flow meter placed in series to control the sampled volume. Sampling was carried out on different days and the same methodology was applied: three tubes of the same type with different volumes (50, 100, and 300 mL) were sampled from the sampling point with a sample rate of 50 mL min^−1^. For a further comparison of the different materials tested, the results obtained were used to calculate and compared total volatile silicon (TVS) in accordance with the Equation (1) edited by Paolini et al. [4]:

(1) $$C_{Si}=\frac{AW_{Si}}{V}\;\sum_{i}\frac{m_{i\;}n_{i}}{MW_{i}}$$

where *C_Si_* is total volatile silicon concentration, *AW_Si_* is the atomic weight of Si (28.0855 Da), *V* is the sampled volume, *m_i_* and *MW_i_* are the measured amount of the individual siloxane and its molecular weight, respectively, and *n_i_* is the number of Si atoms of the individual siloxane.

## 3. Results and Discussion

### 3.1. APCF Efficiency in Thermal Desorption

**Figure 2:**
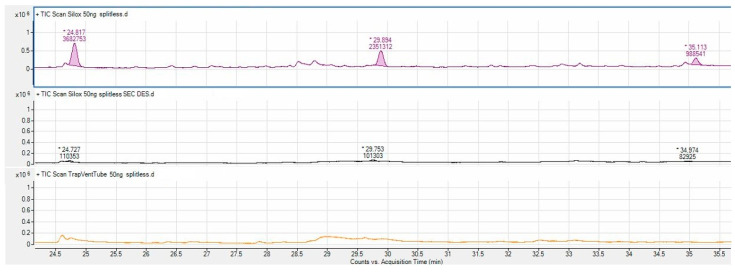
Chromatogram comparison of test at 50 ng. (**Above**) The first desorption with the three peaks D4, D5, and D6. In the (**middle**), the residue in the second desorption. (**bottom**) The result of the backup tube.

Based on the tests conducted to evaluate the efficiency of APCF as an adsorbent material for the analysis of siloxanes, the material was proven to be extremely performing. Specifically, for almost all concentrations of tested standards, the tube and focusing trap desorption was completely quantitative and did not give rise to residues between one desorption and the next. In addition, the backup tubes always produced lower results than the LOD which demonstrates the adsorbent capacity of the focusing trap in APCF. The sampling at higher standard quantities (50 ng) was the only exception. In this case, the second desorption of the tube showed traces of the analytes, but the backup tube still showed lower results than the LOD. Chromatograms are shown in Figure 2, whereas the areas obtained in the first and second desorption and their memory effect in percentage are reported in Table 4. It is good to keep in mind that these are extremely high concentrations for real sampling which is usually based on volumes of hundreds of mL for thermal desorption tubes and therefore hardly reaches 50 ng or even more. However, the memory effects are all less than 10% of the sample and it is sufficient to expect to lengthen the desorption time of the tube and focusing trap on the operating parameters to overcome this problem.

**Figure 3:**
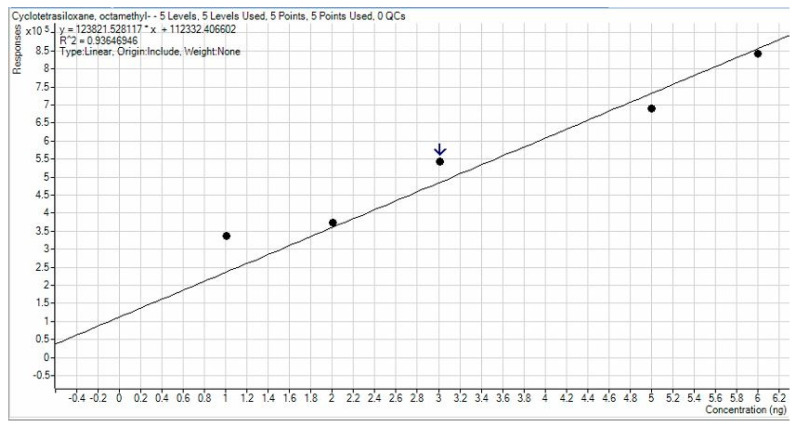
Calibration line for D4.

**Figure 4:**
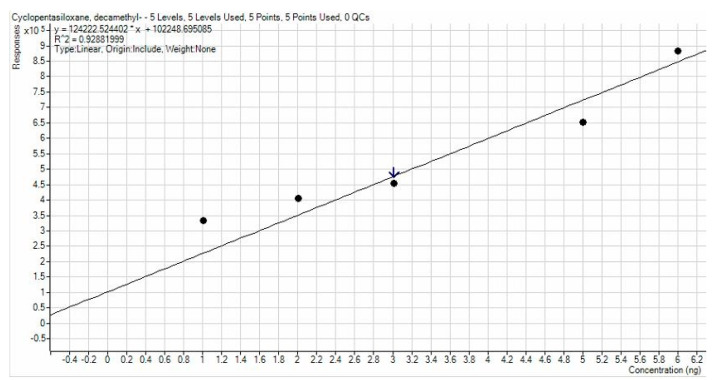
Calibration line for D5.

**Figure 5:**
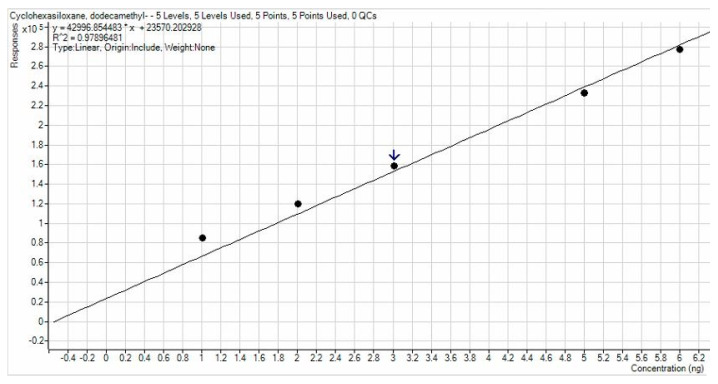
Calibration line for D6.

Calibration lines calculated by dot interpolation gave R^2^ values equal to 0.93647 for D4, 0.92882 for D5, and 0.97896 for D6. Calibration line images are shown in Figure 3, Figure 4 and Figure 5. A higher accuracy was observed for D6, while in other cases, a small deviation of the points from the calibration line was observed for points at lower concentration. The lines obtained were satisfied with a high R^2^ and consequently it is assumed that the results have a high degree of precision and accuracy.

### 3.2. Biogas Sampling

The sampling on biogas plant produced the results shown in Table 5.

It may be not excluded that differences in concentrations could be due to the fact that samplings with standard tubes and APCF were conducted on different days. Moreover, this could also be the consequence of the fact that the standard tubes were desorbed at a temperature of 320 °C unlike the APCF tubes, which were desorbed at 365 °C temperature to avoid disturbing the material. In fact, if the structure and composition of the adsorbent is not affected, higher temperatures allow better quantitative desorption and such phenomena could be the cause of the higher concentrations that lead to a more indicative result of the nature of biogas/biomethane. In any case, it is quite evident that sampling and analysis with APCF had a vastly greater accuracy of the result, with much lower standard deviations than tests conducted with standard multilayer tubes.

TVS concentration calculated in accordance with Equation (1) with APCF and traditional multilayers tube was 0.292 and 0.0875 mg/Nm^3^, respectively. Although both are below the limit of 2 mg/m^3^ reported in the introduction and related to CHP plants, there is a significant difference between the two calculated results, which demonstrates the importance of using an effective sampling and analysis device.

### 3.3. SEM Analysis

**Figure 6:**
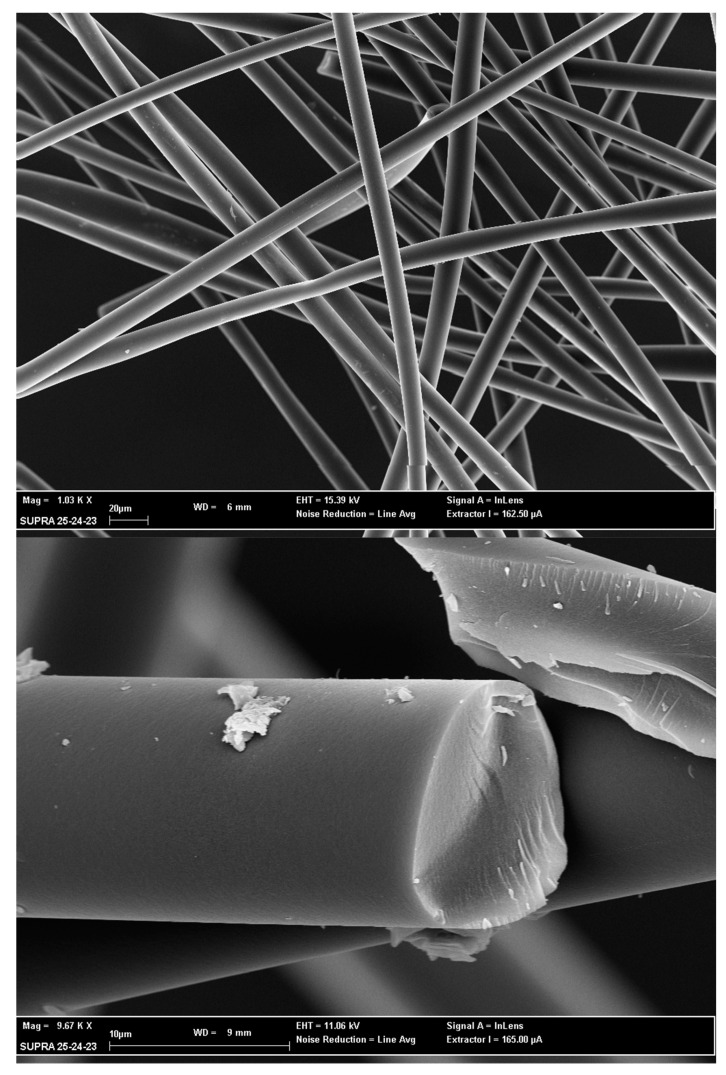

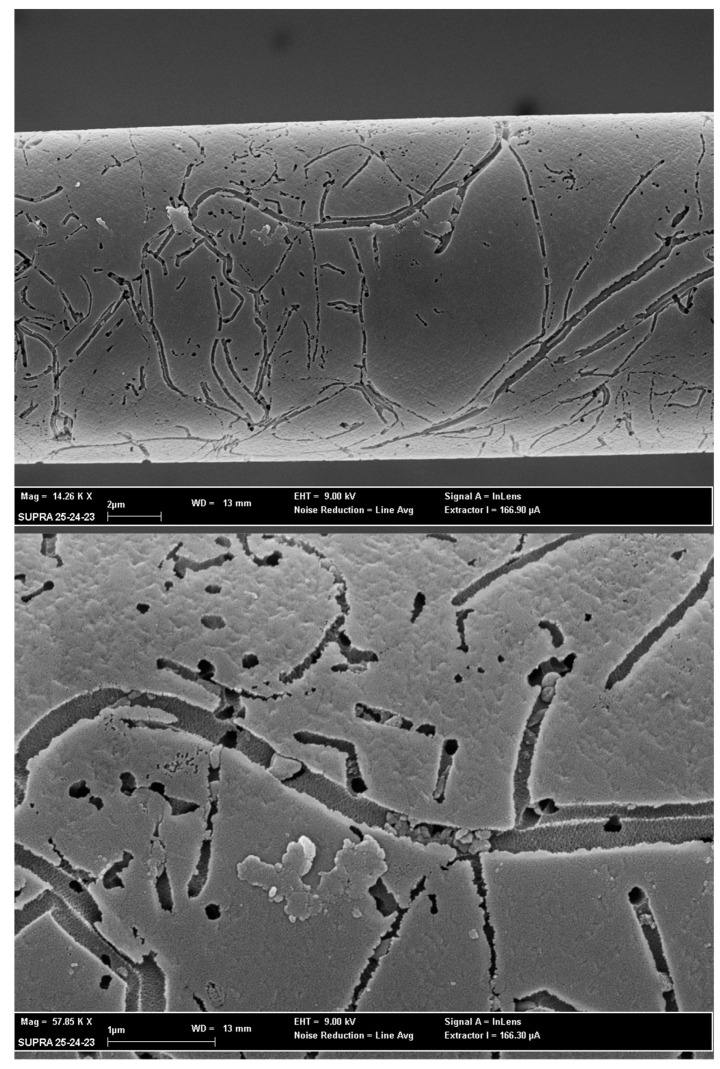
APCF morphological analysis with a magnification range between 1 and 58 K×.

Figure 6 shows the morphological analysis of APCF with a magnification range between 1 and 58 K×. It is evident that the surface of each fiber is sprayed with porous beds rich in macropores within which it is highly probable that there are additional meso and micro pore burrows, typical structure of activated carbons materials.

The results obtained show that APCF is an adsorbent material with a greater porosity and surface development than the common materials used for thermal desorption. In fact, comparing such product with other materials commonly used for the production of thermo-desorption tubes on the market (Tenax, granular activated carbon, etc.), it shows an extremely higher surface area (≈2000 m^2^ g^−1^) which allows to have a high adsorption capacity. Furthermore APCF, as all activated carbon fibers, have the advantage over granular materials that they can be packaged more evenly in the sampling tubes, thus avoiding the presence of preferential routes for analytes during sampling and under-time analysis. It also resists higher temperatures than other commonly used materials, which allows it to provide superior performance during thermal desorption. Without reducing adsorption function, it can be easily regenerated so that it can be reused several times [36]. To the best knowledge of the authors, similar works do not exist in the literature, as the material investigated is innovative. It would be useful to broaden the field of research to other types of siloxanes (such as linear siloxanes) or to other biogas contaminants (such as VOCs) that can be sampled and analyzed by thermal desorption.

## 4. Conclusions

An innovative adsorbent material APCF was tested for its use in biogas sampling and in thermal desorption analysis. Specifically, it was applied to the analysis of VMS (volatile methyl siloxanes) which are among the contaminants that require greater attention during the production and use of biogas and biomethane. Such material is particularly versatile for tubing and focusing traps for thermal desorption and was proven to be optimal using a standard composed of octamethylciclotetrasiloxane (D4), decamethylcyclopentasiloxane (D5), and dodecamethylcyclohexasiloxane (D6). Then the tests conducted on real biogas production plant comparing tubes in APCF and classic multilayers tubes (Carbograph 1TD + Carboxen 1003) showed that APCF provides better results with lower standard deviations when compared to others even with extremely different sampling volumes (50, 100, and 300 mL). APCF being a fiber allows to pack the tubes more homogenously for sampling than the current activated granular carbons, and it is also able to withstand higher T. All this results in a superior adsorption/desorption capacity and, as shown in the results, to more precise and accurate analytical data, both in lab scales with standards and in a real scale with biogas plant-conducted sampling. It is clear that APCF is an interesting adsorbent material for a more accurate monitoring and analysis of biogas and biomethane contaminants and potentially a wide range of volatile compounds.

### Specific and Practical Suggestions

The use of APCF allows to obtain more reliable data, it has been observed how samples at different volumes have little influence on the final analytical data (low standard deviation compared) to traditional tubes. The adsorbent material is water-resistant, like all adsorbent tubes. It is therefore useful to sample downstream of a water removal system or to counterflow the tube immediately after sampling with air (the so-called backflush). Siloxanes are very stable compounds, therefore after sampling there are no particular storage conditions of the sampled tube. However, it is possible that in case the sampling and analysis is extended to other more reactive analytes (e.g., VOC), sampled tubes at low T should be retained and analyses should be carried out within one week of the date of sampling.

## Figures and Tables

**Table 1:** Main chemical-physical characteristics of the siloxanes D4, D5, D6 [3].

Abbreviation	D4	D5	D6
Name	Octamethylcyclo-Tetrasiloxan	Decamethylcyclo-Pentasiloxane	Dodecamethylcyclo-Hexasiloxane
Structure	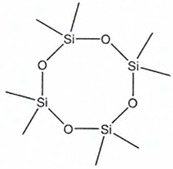	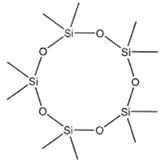	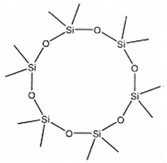
Molecular Formula	C_8_H_24_O_4_Si_4_	C_10_H_30_O_5_Si_5_	C_12_H_36_O_6_Si_6_
Physical Properties	Liquid, colorless, oily, odorless	Liquid, oily	Liquid, colorless, faint odor
Molecular weight (Da)	296.61	370.80	444.93
Boiling point (°C)	175.5	210	245
Melting point (°C)	17.5	7.5	−3
Water solubility (mg/Lw 23 °C)	0.056	0.017	0.005
Molar Volume (g/cm^3^ at 20 °C)	309.2	386.5	463.8
Density (g/cm^3^) at 20 °C	0.953	0.955	0.959
Critical T (°C)	313.35	346.05	382.25
Critical P (atm)	13.2	11.5	12.9
Critical V (m^3^/kmol)	979.0	1216.0	1493.1

**Table 2:** Parameters used for thermal desorption-gas chromatography-mass spectrometry (TD-GC-MS).

Operative Parameters		
**TD**	Desorption time	10 min
	Desorption flow	60 mL min^–1^
	Desorption temperature	365 °C
	Focusing trap temperature	−15 °C
	Focusing trap desorption temperature	370 °C
**GC/MS**	Carrier gas	He
	Column	DB 502.2
	Flow	1.2 mL min^–1^
	GC mode	constant flow
	Oven ramp	35 °C (5 min) + 5 °C min^–1^ to 230 °C (5 min)
	Ion source	EI
	Inlet temperature	200 °C
	MS source temperature	230 °C
	Transfer line Temperature	240 °C
	MS mode	Full Scan 35–450 *m/z*

**Table 3:** Characteristics of adsorbent material used.

	Carbon Fiber Continent (%)	Fiber Diameter (μm)	Specific Surface Area (m^2^ g^–1^)	Density (g cm^–3^)
ACPF ^1^	100	10	≈2000	0.095

^1^ APCF: Activated porous carbon fiber.

**Table 4:** Areas of D4, D5, and D6 peaks in the first and second desorption and their memory effect in percentage.

	I Desorption Test	II Desorption Test	Residue %	Backup Tube
**D4**	3,682,753	110,353	3.0	<LOD
**D5**	2,351,312	101,303	4.3	<LOD
**D6**	988,541	82,925	8.4	<LOD

**Table 5:** Biogas sampling results: comparison between APCF and standard tube (ng mL^−1^).

APCF	D4	D5	D6
**Average**	0.182	0.488	0.101
**Standard Deviation**	0.030	0.083	0.009
**Multilayers Tube**	**D4**	**D5**	**D6**
**Average**	0.076	0.035	0.120
**Standard Deviation**	0.010	0.034	0.112

## Data Availability

All data will be made available on request.
